# Higher body weight and distant metastasis are associated with higher radiation exposure to the household environment from patients with thyroid cancer after radioactive iodine therapy

**DOI:** 10.1097/MD.0000000000007942

**Published:** 2017-09-01

**Authors:** Sheng-Fong Kuo, Tsung-Ying Ho, Miaw-Jene Liou, Kun-Ju Lin, Ru-Chin Cheng, Sheng-Chieh Chan, Bie-Yui Huang, Soh-Ching Ng, Feng-Hsuan Liu, Hung-Yu Chang, Sheng-Hwu Hsieh, Kun-Chun Chiang, Huang-Yang Chen, Ta-You Lo, Chih-Lang Lin, Jen-Der Lin

**Affiliations:** a Department of Endocrinology and Metabolism, Chang Gung Memorial Hospital, Keelung; b Chang Gung University, College of Medicine; c Department of Nuclear Medicine; d Department of Endocrinology and Metabolism, Chang Gung Memorial Hospital, Kwei-Shan, Taoyuan; e Department of Nuclear Medicine; f Department of General Surgery; g Department of Gastroenterology, Chang Gung Memorial Hospital, Keelung, Taiwan.

**Keywords:** radiation exposure, radiation protection, radioactive iodine, rhTSH, thyroid cancer

## Abstract

There were insufficient data regarding radiation exposure to the household environment from patients with thyroid cancer who received radioactive iodine (RAI) therapy in Asia; we therefore performed the present study at the Chang Gung Memorial Hospital in Keelung, Taiwan.

Patients with papillary or follicular thyroid cancer who received 3.7 GBq (100 mCi) RAI were enrolled in this prospective hospital-based study. The enrolled patients were asked to place a thermoluminescent dosimeter in the living room, bedroom, and bathroom of their houses for 4 weeks to measure radiation exposure to the household environment.

A total of 43 patients (18 men and 25 women; mean age 51 ± 13 years) who received 3.7 GBq (100 mCi) RAI completed the study. The mean value of total radiation exposure over 4 weeks from the patients to the bedroom, bathroom, and living room (eliminating the background radiation factor) was 0.446 ± 0.304 (0.088–1.382) mSv. We divided the patients into 2 groups: those with more than and less than the mean value of total radiation exposure to the bedroom, bathroom, and living room. Factors associated with the higher amount of radiation exposure from the patients to the household environment were patient body weight (*P* = .025, univariate analysis; *P* = .037, multivariate analysis, odds ratio [95% confidence interval] 1.067 [1.004–1.134]) and distant metastases based on ^131^I post-therapy scanning (*P* = .041, univariate analysis; *P* = .058, multivariate analysis, odds ratio [95% confidence interval] 6.453 [0.938–44.369]); age, sex, body mass index, renal function, serum stimulated thyroglobulin level, and recombinant human thyroid-stimulating hormone use were not associated with the amount of radiation exposure from the patients to the household environment.

Higher body weight and distant metastases may be the best predictors for higher radiation exposure to the household environment from patients with thyroid cancer after RAI therapy.

## 1 Introduction

Radioactive iodine (RAI) therapy has been extensively used for more than 60 years in the treatment of differentiated thyroid cancer (DTC) after total or near-total thyroidectomy, and is suggested for remnant ablation in high and intermediate-risk patients with DTC and for recurrent DTC.^[1]^ The US Nuclear Regulatory Commission (NRC) Patient Release Criteria allow outpatient ^131^I treatment for most patients.^[2]^ Measurements have demonstrated that radiation exposure within the home did not exceed regulations in comparable studies performed in the United States,^[3]^ Canada,^[4]^ and Brazil^[5]^ when outpatients treated with high-dose RAI for thyroid cancer and their families were instructed in radiation safety. However, the Atomic Energy Council in Taiwan has adopted Title 10 of the Code of Federal Regulations (10 CFR 35.75) revised in 1997 by the US NRC^[6]^ and establish the administration of 1.1 GBq (30 mCi) or higher ^131^I dose as a threshold for inpatient treatment because Taiwan is among the most crowded places in the world. Patients should be isolated in an ^131^I ward and cannot be released until the dose rate is below 50 μSv/h detected from a distance of 1 m.

The American Thyroid Association published practice guidelines for radiation safety in the treatment of patients with thyroid diseases by RAI in April 2011 for patients, their family members, and the public regarding radiation safety,^[7]^ although the guidelines are not completely evidence-based since there are not enough data on long-term outcomes on which to base the use or lack of use of these recommendations. The best we could do was to follow these recommendations to reduce radiation exposure to the greatest possible extent. Considering that there were still insufficient data regarding radiation exposure to the household environment from patients with thyroid cancer who received RAI therapy, and also the factors associated with radiation exposure from the patients to the household environment, we therefore performed the present study at the Chang Gung Memorial Hospital in Keelung, Taiwan.

## 2 Methods

### 2.1 Study population and data collection

In this prospective hospital-based study, subjects were enrolled from among patients with papillary or follicular thyroid cancer who were administered more than 1.1 GBq (30 mCi) of RAI at the Chang Gung Memorial Hospital, Keelung, Taiwan from April 2012 to March 2014. All study participants had to be isolated in the^131^I ward at the Chang Gung Memorial Hospital. The Chang Gung Memorial Hospital Institutional Review Board (IRB) approved the study (No. 101–0188B). Confidentiality of the research subjects was maintained in accordance with the requirements of the IRB of the Chang Gung Memorial Hospital, and all research was conducted in accordance with the principles of the Declaration of Helsinki. Written informed consent was obtained from all study participants before their enrollment. All patients underwent total or complete thyroidectomy. The term “total thyroidectomy” refers to total or near-total thyroidectomy with or without central compartment and selective bilateral neck lymph node dissection. A low iodine diet for approximately 2 weeks before treatment was suggested for patients undergoing RAI therapy. Thyrotropin stimulation or thyroxine withdrawal was performed before RAI therapy. If thyroxine withdrawal was planned before RAI therapy, thyroid hormone (LT4) was withdrawn for 4 to 6 weeks. If thyrotropin stimulation was performed, recombinant human thyroid-stimulating hormone (rhTSH) (thyrogen 0.9 mg) was administered in a course of 2 consecutive daily injections, and RAI therapy was administered 24 hours after the second dose of rhTSH.

All patients in the study had a stimulated TSH >48 mIU/L at the time of RAI therapy. Patient characteristics were recorded, including age, sex, body weight, height, tumor size, serum stimulated thyroglobulin (Tg) level at diagnosis, and serum stimulated Tg level before RAI treatment in this study, and renal function. Tg was detected with an immunoradiometric assay (IRMA) kit (CIS Bio International, Gif-sur-Yvette, France). The detection limit of the Tg kit is 0.5 ng/mL, and its functional sensitivity has been assessed in our laboratory to be 1.2 ng/mL. All the patients were asked to drink at least 2 to 3 L of water for faster RAI elimination after treatment. Patients with heavy weight were not asked to drink more water in this study. All patients were discharged after a stay of 2 days in the ^131^I isolation ward, and the dose rate was less than 50 μSv/h detected from a distance of 1 m. The patients were instructed to remain at home and avoid public places for 1 week after discharge from the hospital. Patients were provided Radiation Safety Instructions upon discharge, and received instructions to stay more than 1 m away from their adult family members and caregivers, and to stay more than 3 m away from infants, young children, and pregnant women. The patients were directed not to share food, toothbrushes, towels, spoons, forks, chopsticks, glasses, or dishes with others. They were asked to flush the toilet 3 times after each use, and men were instructed to sit down to urinate and to keep the bathroom clean. On the 8th day after RAI treatment, the patients returned to the hospital and underwent whole body scanning. The results of the whole body scan were recorded as local (neck area), distant (other than neck area), and negative uptake of ^131^I.

The enrolled patients were asked to place a thermoluminescent dosimeter (TLD) in the living room, bedroom, and bathroom of their houses for 4 weeks to measure radiation exposure upon the patients’ discharge. We used TLDs 7776, which were the commercial product made by Thermo Harshaw. One dosimeter card consisted of 4 TLD chips. The dosimeters were read by the reader 8800 plus in the Institute of Nuclear Research,Taiwan. A total of 52 patients completed the study with the reading of the TLD after their completion of RAI therapy. Forty-three patients received 100 mCi (3.7 GBq), 6 patients received 70 mCi (2.6 GBq), and the other 3 patients received 150 mCi (5.6 GBq) RAI. At first, we would like to include all the 52 patients in this study. Finally, we included 43 patients undergoing 3.7 GBq (100 mCi) RAI and excluded those treated by 2.6 GBq (70 mCi) (6 patients) and 5.55 GBq (150 mCi) (3 patients) to keep data consistency and to avoid unnecessary confounding factor. Among the 43 included patients who underwent 3.7 GBq (100 mCi) RAI, there were 25 women and 18 men with a mean age of 51 ± 13 years.

### 2.2 Measurement and analysis of radiation exposure

Before receiving RAI therapy, the enrolled patients were also asked to place a TLD in the living room, bedroom, and bathroom of their houses for 4 weeks to measure background radiation exposure. The TLDs were placed about 1 m from the sites of the patients’ usual activities and were retrieved 4 weeks later and sent to the Institute of Nuclear Energy Research (INER) in Taiwan for analysis. The patients were then admitted to the isolation ward at the Chang Gung Memorial Hospital, Keelung, Taiwan. TLDs were given again to their families to place in the same sites in the living room, bedroom, and bathroom upon the patients’ discharge. TLDs were subsequently retrieved another 4 weeks later and sent to the INER in Taiwan for analysis of the dose of accumulated radiation exposure. With subtraction of the background radiation exposure from the accumulated radiation exposure, the absolute values of radiation exposure from the patients could be determined.

Factors that might be associated with radiation exposure to the household environment were analyzed, such as age, sex, tumor size at diagnosis, serum creatinine level, serum stimulated Tg level, and results of post-therapy scanning. Analysis of differences between the thyroxine withdrawal and rhTSH groups was also performed. Renal function was assessed by the Modification of Diet in Renal Disease (MDRD): MDRD—Simplify—GFR (mL/min/1.73 m^2^) = 186 × Scr − 1.154 × Age − 0.203 × 0.742 (if female).

### 2.3 Statistical analysis

Discrete data are reported as absolute frequency and percentages, and compared using chi-square or Fisher exact tests, where appropriate. Continuous data are reported as means and standard deviation (SD), and compared using the Student *t* test or Mann–Whitney *U* test, where appropriate. To evaluate the relationship between the amount of radiation exposure to the household environment and basic clinical characteristics, the continuous data were dichotomized into 2 groups, with the means as cut-offs. Univariate and multivariate logistic regression analyses were performed to reveal the factors associated with the amount of radiation exposure. All tests were 2-tailed, and a *P* value of <.05 was considered statistically significant. All statistical analyses were performed using the Statistical Package for the Social Sciences (SPSS) for Windows Version 15.0 (SPSS Inc., Chicago, IL).

## 3 Results

### 3.1 Patients

There were 38 patients with papillary thyroid cancer and 5 patients with follicular thyroid cancer. Table 1 shows the basic characteristics of the 43 patients, including body weight, height, body mass index (BMI), tumor size at diagnosis, soft tissue involvement or distant metastasis at diagnosis, previous accumulated ^131^I treatment dose, renal function, rhTSH preparation before RAI treatment or not, stimulated Tg after surgery, stimulated Tg before RAI treatment in this study, and post-therapy whole body scan results. Twenty-seven patients were receiving their initial RAI therapy for remnant ablation, whereas the other 16 patients had received RAI therapy more than once for recurrent thyroid cancer with a previous accumulated ^131^I dose of 11.3 GBq (305.3 mCi) (1.1–12.95 GBq [30–350 mCi]). At 2 days post-therapy, the patients were discharged from the hospital with a median emission at a distance of 1 m of 17 μSv/h. Post-therapy whole body scanning on the 8th day after administration of RAI therapy revealed distant (other than neck) ^131^I uptake in 7 patients (16.3%), local (neck) uptake in 31 patients (72.1%), and negative uptake in 5 (11.6%) patients. Of the 7 patients with distant metastasis based on post-therapy scanning, 4 patients had ^131^I uptake in the lung, 2 had ^131^I uptake in the mediastinum, and 1 had ^131^I uptake in the bone.

**Table 1:**
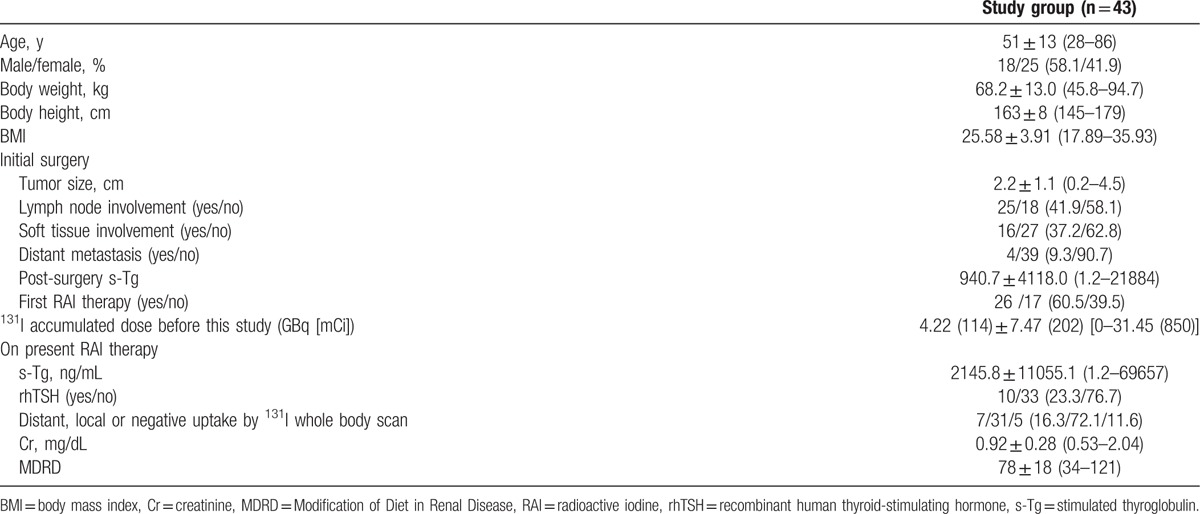
Basic characteristics of the 43 patients receiving 3.7 GBq (100 mCi) ^131^I treatment.

### 3.2 The amount of household radiation exposure

**Figure 1:**
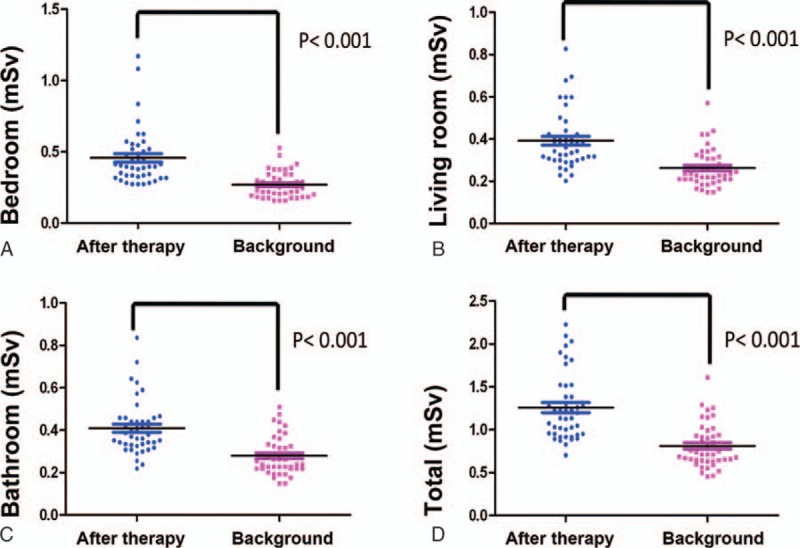
Higher levels of radiation exposure to household environment after patients underwent radioactive iodine therapy compared with those from background radiation exposure.

The accumulated dose of radiation exposure over 4 weeks after the patients underwent RAI therapy was 0.4566 ± 0.1961, 0.3922 ± 0.1362, and 0.4094 ± 0.1265 mSv in the bedroom, the bathroom, and the living room, respectively, whereas the accumulated dose of background radiation exposure for 4 weeks was 0.267 ± 0.0903, 0.263 ± 0.0887, and 0.2793 ± 0.0896 mSv in the bedroom, the bathroom, and the living room, respectively (Table 2). There was significantly higher radiation exposure to the household environment after the patients underwent RAI therapy compared with that from background radiation exposure (*P* < .001) (Fig. 1). The absolute increase in radiation exposure from the patients for 4 weeks after RAI therapy (eliminating the effect of background radiation) was 0.1866 ± 0.162, 0.1292 ± 0.1205, and 0.1301 ± 0.1061 mSv in the bedroom, the bathroom, and the living room, respectively (Table 2). Thus, the absolute increase in total radiation exposure from the patients to the bedroom, the bathroom, and the living room was 0.446 ± 0.304 (0.088–1.382) mSv in 4 weeks.

**Table 2:**
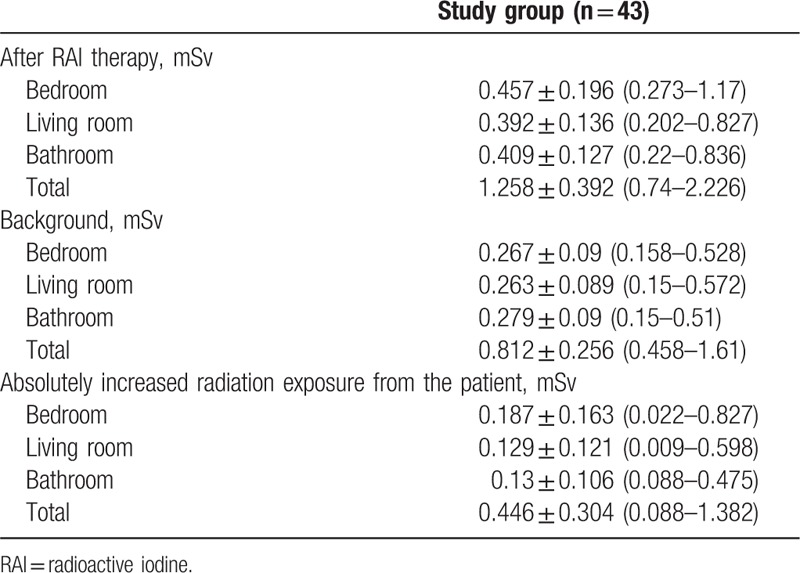
Radiation exposure from the patients to the household environment.

### 3.3 Factors associated with the amount of radiation exposure

According to the amount of radiation exposure to the household environment, we divided the patients into 2 groups (>0.446 mSv and <0.446 mSv; ie, more than and less than the mean value of total radiation exposure to the bedroom, bathroom, and living room) (Table 3). No factors including age, sex, BMI, renal function (including serum creatinine level and MDRD value), rhTSH preparation, tumor size at diagnosis, serum-stimulated Tg after surgery, or stimulated Tg before RAI therapy were associated with the amount of radiation exposure from the patients to the household environment in the present study. Whether the patients had received their initial RAI therapy and the^131^I accumulated dose before the study were also not associated with the amount of radiation exposure to the household environment. In contrast, factors significantly associated with the amount of radiation exposure from the patients were patient body weight (*P* = .025, univariate analysis; *P* = .037, multivariate analysis; odds ratio [OR] 1.067, 95% confidence interval [CI] 1.004–1.134) and distant metastases diagnosed by ^131^I post-therapy scanning (*P* = .041, univariate analysis), although the results of multivariate analysis for distant metastases were not significant (*P* = .058, multivariate analysis; OR 6.453, 95% CI 0.938–44.369). Higher body weight and distant metastasis were associated with higher radiation exposure to the household environment from patients with thyroid cancer after RAI therapy. In addition, we specifically measured the absolute amount of radiation exposure from male and female patients to the bathroom after eliminating the effect of background radiation exposure, and the results showed that there was no difference in radiation exposure to the bathroom between men and women (0.1462 ± 0.1237 vs 0.1184 ± 0.0923 mSv, respectively; *P* = .404).

**Table 3:**
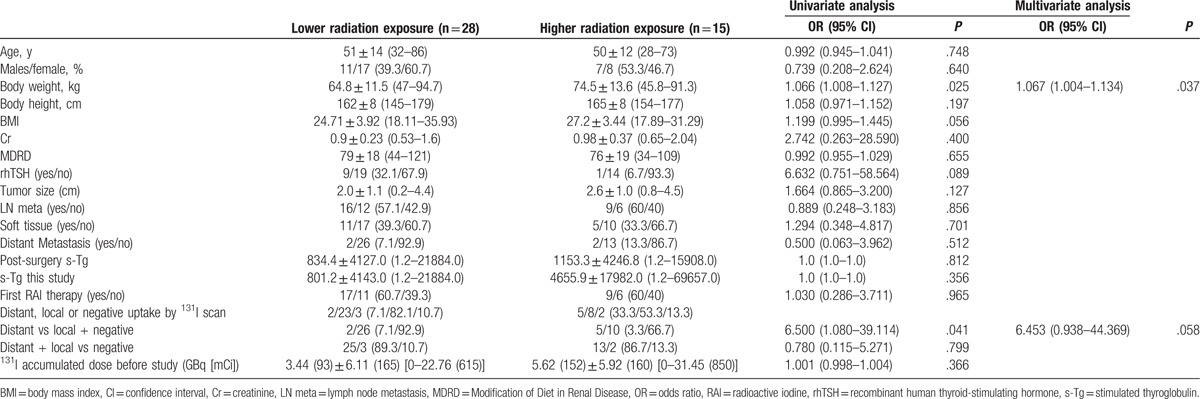
Factors associated with higher radiation exposure (greater than mean value 0.446 mSv) from patients to the household environment.

## 4 Discussion

Radioactive iodine therapy has been used in the treatment of patients with papillary or follicular thyroid cancer since the 1940s, and it is usually administered to these patients after thyroidectomy for remnant ablation and treatment of metastatic thyroid cancer. Patients who receive RAI have the potential to expose their family to low levels of radiation via saliva, urine, or radiation emitting from their bodies. Although harm due to radiation exposure from patients treated with ^131^I has not been demonstrated, our hospital staff follow the principle of reducing radiation exposure to levels that are as low as reasonably achievable (ALARA), and acknowledge that even unapparent radiation injuries are cumulative, and that, over time, small effects contribute to definitive risks.^[7]^ In a study of patients with thyroid cancer who received 2.8–5.6 GBq (75–150 mCi) of RAI as outpatients, exposure of family members was minimal when precautions were followed,^[3]^ results compatible with those of other later studies conducted in Canada and Brazil.^[4,5]^ Radiation exposure to caregivers after the patient is quarantined in the hospital for 3 to 4 days may be even lower.^[8,9]^ Since Taiwan is among the most highly populated countries in the world, the results of our data provide useful information for patients with thyroid cancer receiving high-dose RAI therapy, and add to our knowledge regarding radiation safety for our environment.

Although it is possibly right that the given RAI dose to the patients was proportional to the amount of RAI retention in patient's body or the amount of radiation exposure from the patients, the absorption of RAI is different from patients to patients and would be interfered by many biologic factors including serum TSH level, iodine content in the food, absorptive rate of the intestine, and so on.^[10]^ Therefore, we analyzed only the data of 43 patients undergoing 3.7 GBq (100 mCi) RAI, and excluded those treated by different RAI dose. The results of the present study revealed that higher patient body weight, but not higher BMI, was associated with higher radiation exposure to the household environment from patients with thyroid cancer after RAI therapy, which may indicate that heavy weight patients could emit more radiation upon other individuals after RAI therapy, and the family or other accompanying individuals might require greater radiation protection from these heavyweight patients. In fact, it is probable that the higher radiation exposure of higher body weight patients could be due to their higher dose rate at time of hospital discharge, so we split the patients with higher radiation exposure (n = 15) into 2 groups of higher or lower body weight based on the average body weight. Patients with higher body weight had the same external dose rates (16.2 ± 5.3 uSv/h, n = 9) at time of hospital discharge as those with lower body weight (20.8 ± 4.9 uSv/h, n = 6) (*P* = .404). Thus, the higher radiation exposure of higher body weight patients was not associated with their higher dose rate at time of hospital discharge. Although there is no literature addressing the association of patients’ body weight and the radiation exposure, we think that higher body weight might be associated with larger body volume, which could retain more amount of RAI in the body and subsequently more radiation exposure to the environment. Moreover, patients who were found to have distant metastases based on post-therapy scanning, for example, ^131^I uptake in lung, mediastinum, or bone, were more likely to expose their families to greater amounts of radiation.

Radioactive iodine uptake by the thyroid is stimulated by TSH. There are 2 methods for increasing TSH: thyroid hormone withdrawal or administration of rhTSH. Some publications have reported lower residual radiation in patients with the use of rhTSH.^[11,12]^ The total body radiation exposure in patients prepared with rhTSH is lower than that in those with thyroid hormone withdrawal and might be due to the more rapid clearance of RAI from euthyroid versus hypothyroid states. In addition, administration of rhTSH was associated with statistically significantly longer remnant half-life and shorter remnant residence time of RAI (but not statistically significant) than thyroid hormone withdrawal^[12,13]^; however, the effect of rhTSH on the residence time of the metastatic lesions was still not clear. In the present study, however, we did not find lower amounts of radiation to the household environment emitting from the patients treated with ^131^I who were prepared with rhTSH. This finding may indicate that other factors played more important roles than did rhTSH in radiation exposure to the household environment from the patients after 2 days of quarantine in the hospital. This result is compatible with that of another study which showed the mean radiation dose delivered to family members was similar with either rhTSH or thyroid hormone withdrawal.^[14]^ In addition, we noted that renal function was also not a critical factor for the amount of radiation exposure to the household environment from patients with thyroid cancer after RAI therapy. Furthermore, whether the patients were receiving their initial RAI therapy or not was not associated with the amount of radiation exposure from the patients to the household environment, although there could possibly be more radiation exposure from patients receiving their first RAI therapy due to greater ^131^I uptake of the remnant after surgery.

The amount of time patients spent in the bedroom was presumed to be the longest, so the radiation exposure in the bedroom from the patients was about 50% higher than the exposure in the bathroom and the living room. Urine is the primary excretion route for ^131^I; thus, urine from the patients may contain RAI and contaminate the environment. The amount of time the patients spent in the bathroom was presumed to be the shortest, but the radiation exposure in the bathroom may increase due to the lack of temporary storage facilities for urine in the patients’ houses. Although there were no evidence-based data regarding greater radiation exposure via urine from men, they were presumed to create more splatters of radioactive urine in the bathroom. In the present study, we gave the patients Radiation Safety Instructions upon discharge and directed male patients to sit down to urinate and to keep the bathroom clean, and the results of the study did not show greater radiation exposure from male patients to the bathroom.

The National Council on Radiation Protection and Measurements (NCRP) in the United States suggests that for nonpregnant adults exposed to a family member who is a patient receiving radionuclide therapy, the dose limit is 5 mSv.^[15]^ The dose limit recommended by the International Atomic Energy Agency (IAEA) for the general public is 1 mSv.^[16]^ Based on the US NRC Regulation 10 CFR 35.75, patients released from the hospital must be provided with verbal and written instructions if any individuals are likely to receive doses of more than 1 mSv from the patients.^[17]^ Compared with the above regulations, the average total radiation exposure from the patients to the household environment in the present study was 0.446 ± 0.304 mSv in 4 weeks, which is below the suggested values in the current guidelines. Furthermore, in this study, the radiation exposure from the patients was directed toward the household environment, not directly toward the patients’ families or caregivers. The radiation exposure from the patients toward their families was expected to be lower than that toward the household environment unless the family members were in close contact with the patients continuously for long periods of time after discharge.

This is the first report analyzing factors affecting radiation exposure to the household environment from patients with thyroid cancer treated with ^131^I in Asia, but there were certain limitations in this study. First, we did not know the exact duration of time that the patients spent in the living room, bathroom, and bedroom during the 4-week study. Second, after a stay of 1 week at their house after discharge from the hospital, the patients were not required to remain at home. Furthermore, the small patient population of the study may be insufficient to make robust conclusions. For example, a modest sample of 7 metastatic lesions and 10 patients with rhTSH-assisted ^131^I therapy in this study seems inadequate statistically. Larger series with larger sample sizes are needed to confirm the current conclusions.

## 5 Conclusions

In conclusion, although the radiation exposure to the household environment from patients with thyroid cancer administered 3.7 GBq (100 mCi) RAI was significant compared with background radiation exposure, the impact of the radiation exposure on the patients’ families or caregivers may be small and requires further investigation. Body weight and distant metastases based on post-therapy scanning are the best predictive factors for higher radiation exposure from the patients. Patients with higher body weight or patients with distant metastases based on post-therapy scanning could emit more radiation upon other individuals, and the families or caregivers might require greater radiation protection from these patients.

## Acknowledgments

We are grateful for support from our colleagues in the department of nuclear medicine at Keelung Chang Gung Hospital and the Institute of Nuclear Energy Research (INER) in Taiwan. We also thank the National Science Council in Taiwan for grant support (NMRPG2B0011 & NMRPG2B0012). We also would like to thank Kuo-Wei Lee (INER) for analysis of the dose of accumulated radiation exposure.
